# Endometrial immune profiling and precision therapy increase live birth rate after embryo transfer: a randomised controlled trial

**DOI:** 10.3389/fimmu.2025.1523871

**Published:** 2025-02-24

**Authors:** Nathalie Lédée, Marie Petitbarat, Geraldine Dray, Lucie Chevrier, Alaa Kazhalawi, Mona Rahmati, Eric Vicaut, Abdourahmane Diallo, Nino Guy Cassuto, Lea Ruoso, Laura Prat-Ellenberg

**Affiliations:** ^1^ MatriceLab Innove Laboratory, Immeuble Les Gemeaux, Creteil, France; ^2^ Centre d’Assistance Médicale à la Procréation, Hôpital des Bluets, Paris, France; ^3^ Université Versailles- St Quentin en Yvelines (UVSQ), Unité de Formation et Recherche (UFR), Versailles, France; ^4^ London Women’s Clinic, London, United Kingdom; ^5^ Unité de Recherche Clinique, Hôpital Fernand Widal, (APHP), Université Paris-Diderot Paris 7, Paris, France; ^6^ Laboratoire Drouot, Paris, France

**Keywords:** endometrial immune profiling, uterine immune regulation, precision care, IVF, pregnancy, randomised controlled trial

## Abstract

**Introduction:**

Despite advancements in assisted reproductive treatments, 70% of transferred embryos fail to implant successfully, yielding significant personal and global repercussions. One promising avenue of research is to take into account the individual’s immune uterine profile in order to tailor treatment and optimise outcomes. This randomised controlled trial represents the initial exploration into the consequences of disregarding the state of the uterine immune environment in infertile women embarking on IVF/ICSI.

**Materials and methods:**

This randomised controlled open two-arm trial included IVF patients, with assessment of immune endometrial environment and precision therapy before embryo transfer (ET). 493 patients were enrolled from October 2015 to February2023. Endometrial biopsies were collected during the mid-luteal phase. Endometrial immune profiling involves the analysis of cytokine biomarkers in the endometrium. If an immune endometrial dysregulation was diagnosed, a computerised randomisation assigned patients to either a conventional ET (disregarding the immune profile) or a personalised ET (with a precision therapy adapted to the immune profile). The primary analysis focussed on demonstrating the superiority of precision treatments using the modified intent-to-treat population (mITT), excluding patients without ET. The primary endpoint was the live birth rate (LBR) following the first attempt of ET.

**Results:**

Among the population, 78% had an endometrial immune dysregulation and were randomised. The mITT analysis showed a significant increase in LBR with precision care compared to conventional care (29.7% vs. 41.4%; OR: 1.68 [1.04-2.73], p=0.036). The positive impact of precision care was particularly noticeable in patients with morphologically suboptimal embryos (LBR: 21.2% vs. 39.6%; OR: 2.12 [1.02-4.41]) or those with a history of two or more failed ET (LBR: 23.4% vs. 48.1%; OR: 3.03 [1.17-7.85]).

**Limitations and reasons for caution:**

The data should be interpreted with caution due to inherent structural limitations of human IVF trials. Generalising and empowering our findings would rely on the replication of controlled trials by independent research teams possibly integrating the usage of optimised embryo quality with PGT-A.

**Conclusion:**

The regulation of the endometrial immune environment emerges as one of the leading innovative strategies to facilitate embryo implantation and enhance the overall performance of assisted reproductive therapies (ART). Based on these findings, endometrial immune profiling could become an essential part of routine ART practice.

**Clinical trial registration:**

clinicaltrials.gov, identifier NCT02262117.

## 1 Introduction

Assisted reproductive technologies (ART) have made significant progress in the last few decades and have become a widely accepted therapy for infertility. According to WHO, 15% of couples having unprotected sex, representing 48 million couples and 186 million people, suffer from infertility worldwide (1). However, despite the improvements in ART, the success rates remain relatively low. Indeed, the live birth rate per initiated cycle is approximately 30% for women under 35 years old (equal to 70% of failure) and decreases drastically with age (2). This leads to emotional, psychological, and financial stress with significant social and economic consequences, including loss of productivity and a decline in the quality of life for couples (3). Therefore, there is an urgent need for research and innovation to improve success rates and reduce the emotional and financial burden associated with conventional treatment.

Endometrial immune profiling is an innovative strategy involving the analysis of functional immune biomarkers in the endometrium. This approach aims to identify immune disturbances contributing to embryo implantation failures or pregnancy loss and guide the development of personalised treatment plans to increase embryo implantation rates. Incorporating uterine immunity as a key factor in routine practice for designing effective reproductive treatments has not been undertaken thus far. Human pregnancy is a precisely timed (4) semi-allograft that needs to be tolerated by the maternal immune system to survive in physiological conditions. (5)The maternal immune system itself has to be reprogrammed towards tolerance (6, 7). An increasing number of clinical studies also report the essential role of immune cells in endometrial receptivity to embryo implantation and early placental development (8, 9). The Uterine Immune Profile offers a simplified representation of the complex immune processes involved in implantation. The clinical objective is to create a tool that helps clinicians apply precision medicine by integrating this essential local immune response. Previous extensive cohort studies, focussing on individuals with a history of repeated unexplained implantation failures or unexplained recurrent miscarriages using endometrial immune profiling, have revealed that 75-80% of these infertile patients have uterine dysregulations impeding the implantation process. Personalising care to address observed dysregulations has yielded significant benefits, with a relative increase of 40-50% in live birth rates observed compared to the ones expected in these populations (10–12).

Human implantation involves the synchronised interaction of the embryo and the endometrium. The window of implantation (WOI) defines the crucial time frame of uterine receptivity when the endometrium undergoes changes in response to hormonal signals from the ovary (mainly progesterone), preparing it to receive and support an embryo (i.e. decidualisation) (13). Endometrial immune cells play a critical role in the process, as they contribute to the establishment of a receptive environment for the embryo to implant and develop (14, 15). During this window, a crucial shift from adaptive immunity to innate immunity takes place in the endometrium (16). This shift creates an immunologically tolerant and fruitful environment for the developing embryo, which is a semi-allograft. The balance between Th1 and Th2 cytokines, initially described by Tom Wegmann thirty years ago, plays an essential role in the success of implantation (17). The shift to a Th2-dominant immune environment influences the differentiation of immune cells, including macrophages, dendritic cells, uterine NK cells, and regulatory cells, either positively or negatively, thereby promoting or inhibiting implantation and placentation (7, 18). The quantification of RNA expression levels of five biomarkers gave key information regarding the immunoregulated Th-2/Th-1 local balance, the destabilisation of spiral arteries, and the mobilisation and maturation of the very specific uterine natural killer (uNK) cells (19). Interleukin-18 (IL-18) is a pro-inflammatory cytokine crucial for immune regulation in reproduction, playing key roles in embryo implantation, trophoblast invasion, NK cell modulation, and placental vascularisation (20–22) Interleukin-15 (IL-15) supports embryo implantation and placentation by promoting uterine natural killer cell maturation, function and cytokine production essential for reproductive processes (23–25). In the context of embryo implantation, TWEAK/Fn-14 signalling has been shown to regulate the cytotoxicity of uNK cells, which is important for controlling trophoblast invasion and preventing foetal rejection (26, 27). Hence, the ratio of IL-18/TWEAK mRNA was used as a biomarker that served as an indicator of both angiogenesis and the Th1/Th2 balance. IL-18/TWEAK provided insights into the local immune environment and the potential presence of an immune deviation towards Th1 cytokines, which can affect the implantation process (11). On the other hand, IL-15/Fn-14 mRNA was used as a biomarker to assess the activation and maturation status of uterine natural killer (uNK) cells, along with the evaluation of uNK-CD56 cell count (11). By quantifying these targets, we established the endometrial immune profile during the mid-luteal phase, aiming to understand how the endometrium is prepared for successful implantation and to identify dysregulations that may hinder this process (28).

One promising avenue of research is the use of precision medicine approaches, considering an individual’s unique immune endometrial profile to tailor treatment and optimise outcomes. Specific immune cell biomarkers identified through endometrial profiling can guide the selection of appropriate immune-modulating therapies to improve pregnancy outcomes. By gaining a deeper understanding of the endometrial immune profile, we may be able to offer more effective and individualised care to patients struggling with infertility.

In the present randomised controlled trial (RCT), the endometrial immune profile was performed in IVF patients before their embryo transfer. If a local immune dysregulation was diagnosed, computerised randomisation allocated the patient to either a conventional embryo transfer (disregarding the immune profile) or a personalised embryo transfer (considering the immune profile and a proposed plan to correct the dysregulation). The primary outcome was the live birth rate following embryo transfer among dysregulated patients with conventional versus precision care. This RCT explored the consequences of not considering the endometrial immune environment in patients during IVF treatment.

## 2 Methods

### 2.1 Study design

The study protocol was approved by Institutional Review Boards at the University Paris Diderot (clinicaltrials.gov NCT02262117) and our trial followed the extended CONSORT guidelines.

The study has been designed as an open RCT including infertile patients below 38 years with no ovarian insufficiency involved in assisted reproductive treatment and for which documentation of their immune endometrial environment has been performed before a scheduled embryo transfer.

Patients with a diagnosed endometrial dysregulation were randomised: half of the patients received conventional medical care (disregarding the immune profile) while the other half received precision medical care (according to their immune endometrial profile). The primary analysis was based on the modified intention-to-treat population (excluding patients without ET) and the primary efficacy endpoint was the live birth rate (LBR).

This randomised controlled trial was spread over 10 years because we had to revise our initial approach. From October 2014 to August 2016, we started this RCT with the same inclusion criteria and the same mITT but the randomisation was between endometrial profiling vs. no endometrial profiling, although all patients had a biopsy at enrolment. We decided to abandon this study design because in the case of randomisation to the ‘no endometrial profiling’ arm, we would have missed some important information such as the presence or absence of endometrial dysregulation and its type, which is essential for further analysis. 12 Patients included in the group “no immune profiling” group who had an endometrial biopsy stored but not analysed were re-included in the this RCT. Major amendment was applied for this new design and a new electronic list of randomisation was generated.

The first patient was included on October 30th, 2015, the last patient on February 8th, 2023.

A Data and Safety Monitoring Board (DSMB) reviewed interim results periodically throughout the study. No change was made to the personal treatment design during the study, except enlargement of inclusion criteria. Amendments were applied in 2017 to enlarge criteria of inclusion to a range of three oocytes pick up (initially set a 2) and to accept patients using frozen embryos (initially only fresh ET). This new design required more patients to be included. The protocol was suspended during the COVID-19 pandemic in 2020.

As two authors (NL, MPB) hold the patent related to the presented innovation, measures were implemented to ensure the impartiality of the study and address potential bias. To this end, patient inclusion and follow-up until birth or not were independently inspected by a clinical research associate from the independent clinical research unit of the University of Paris Diderot. Furthermore, statistical analyses were conducted by a statistician from the same independent research unit to ensure objectivity in data interpretation.

All patients were followed at the same ART Unit (“Pierre Rouques Les Bluets” Hospital in Paris, France) for the endometrial biopsy, the oocyte retrieval and the embryo transfer.

**Figure 1:**
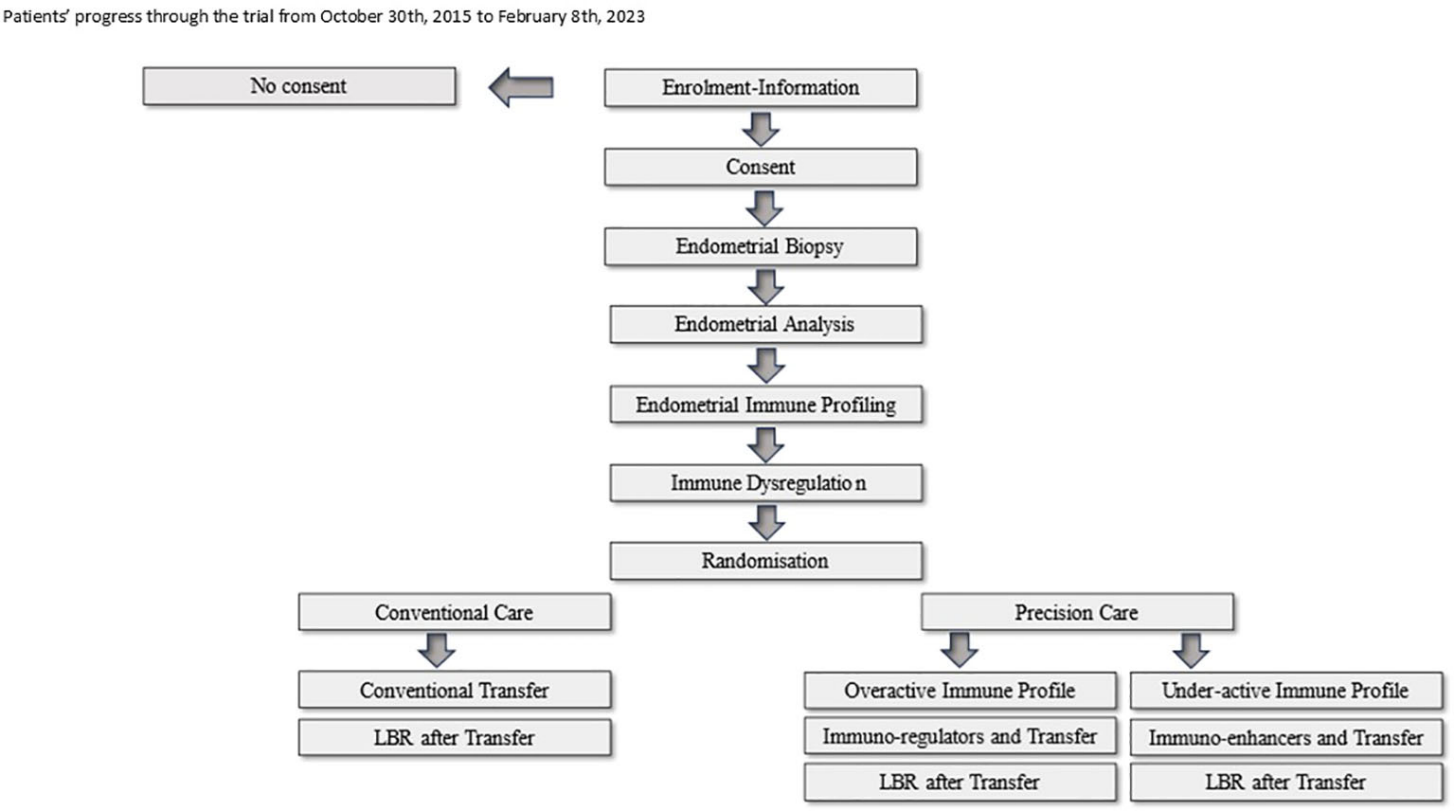
Patients' progress through the trial from October 30th, 2015 to February 8th, 2023.

The study flow is illustrated in **Figure 1**.

#### 2.1.1 Stage 0: consent, inclusion criteria and information

The inclusion criteria were infertile patients with an indication to perform either an IVF with or without ICSI. The indication for IVF were tubal infertility, endometriosis, ovarian dysovulation or idiopathic infertility after IUI failure. The indication for ICSI was male infertility (oligo-astheno-teratospermy) or previous failure of oocyte fertilisation in IVF. Patients were younger than 38 years old (age < or equal to 38 years at the time of inclusion), with no ovarian insufficiency (AMH>1.5ng/ml, FSH<10 IU/l on day-3, antral follicles count (AFC) over 6 on day-3 of the cycle by ultrasound). The range of previous oocyte pick-up for IVF attempts were strictly lower than 3. If a previous live birth had occurred in the past by IVF, the range of the new attempt was 1. Patient had signed an informed consent form and had medical insurance.

The exclusion criteria were azoospermia or cryptozoospermy for the partner, a uterine malformation, an IVF attempt scheduled in another ART unit or contraindication to the use of corticoids, HCG or slow intralipid perfusion.

If the inclusion criteria have been met and exclusion criteria were absent, the clinician informedthe patient of the proposed protocol. If patients agreed to participate, an endometrial biopsy was scheduled in the mild luteal phase.

#### 2.1.2 Stage 1: endometrial biopsy, collection and analysis

In order to target the mid-luteal phase and avoid problems associated with cycle fluctuations, 90% of patients were prepared in a substituted cycle and samples were taken exactly 7 days after the introduction of progesterone.10% were evaluated on a monitored natural cycle and samples were taken 9 days after the LH surge with progesterone dosed 48 hours before sampling. The endometrial fragment was gently aspirated by rotating a Cornier Pipelle within the endometrial cavity (Leclair et al., 2011). The Pipelle content was divided into two parts: the first part was placed in 4% formaldehyde (QPath Formol 4% buffered, VWR Chemicals, Fontenay-sous-Bois, France) for endometrial datation (29), by a histological test to determine the phase of the cycle, and CD-56 immuno-labelling. The second part was placed in RNAlater stabilisation solution for immunological analysis (MatriceLab Innove, France). The samples were sent at room temperature by postal services.

##### 2.1.2.1 RNA extraction and reverse transcription

After confirmation of the histological dating, RNA was extracted from the biopsy sample conserved in RNAlater (Qiagen). RNA extraction was performed on Biomek1.5 using Kit RNAdvance Tissue (Beckman-Coulter). The RNA was reverse-transcribed into cDNA with the First Strand cDNA Synthesis Kit for RT-PCR (Roche, Meylan, France), according to the manufacturer’s instructions. The cDNAs were stored at –20°C until use.

##### 2.1.2.2 Quantitative RT-PCR

Quantitative RT-PCR was performed with a Light Cycler 480 instrument (Roche Diagnostic) and the Light Cycler 480 SYBR Green I Master mix (Roche Diagnostic). Final concentrations for reaction set-up were 0.5 µM of sense and anti-sense primers and 1/20 of diluted cDNA. Cycling conditions were as follows: denaturation (95°C for 5 min), amplification and quantitation (95°C for 10 s, 60°C for 10 s and 72°C for 15 s) repeated 40 times, a melting curve program (65-95°C with a ramp rate of 2.2°C/s) and a cooling step to 4°C. Each quantitative RT-PCR assay included a solution without cDNA and inter-run calibrator (IRC) samples as negative and positive controls. The IRC for all the primers (IL18, IL15, TWEAK, Fn14 and CD56) was obtained from pools of RNA endometrial samples. The IRC cDNA, after dilution by a factor of 20, underwent the same quantitative RT-PCR protocol as the unknown samples. PCR efficiency for each quantified target and reference was calculated with known serial dilutions of each specific cDNA. LightCycler^®^480 Software release 1.5.0 was used to analyse data, and each specific target transcription level was normalised to the geometric mean of the transcription level of the reference gene, with the software’s advanced relative quantification workflow. Gene amplification efficiency was specifically determined. For each sample, the results were expressed as the ratio of target/reference cDNA.

##### 2.1.2.3 Immunohistochemistry (IHC) of uNK cells

IHC was performed on the biopsy sample tissue conserved in 4% formol on 5-µm thick slides, with an automated streptavidin-biotin method (Benchmark GX, Ventana Medical Systems). The prediluted anti-CD56 (clone 123C3) murine monoclonal primary antibody (Ventana Medical Systems^®^, Roche Diagnostics) was applied according to the manufacturer’s instructions. Briefly, after deparaffinization of the slides, antigen retrieval was performed for 60 minutes in a pH 8.4 Cell Conditioning 1 solution. The CD56 primary antibody was then applied for 32 min. Slides for negative controls were prepared by replacing the primary antiserum with non-immune IgG. Slides were then incubated for 8 min with a biotinylated anti-mouse secondary antibody. Diaminobenzidine or 3-amino-9-ethylcarbazole was used as the chromogen (iVIEW DAB detection kit, Ventana Medical Systems) and slides were counterstained with haematoxylin for 2 min, incubated in bluing reagent (for 2 min), and mounted. Between each step, slides were rinsed with reaction buffer. The uNK cell count was measured as the mean of CD56+ cells in 4 representative fields at ×400 magnification.

To establish the endometrial immune profile, a step‐by‐step procedure first considered the IL-18/TWEAK mRNA ratio (reflecting local angiogenesis and possibly a Th1 deviation), then the CD56+ cell count (reflecting uNK cell mobilisation), and finally the IL‐15/Fn‐14 mRNA ratio (indicative of uNK cell maturation and uNK cytotoxic activation).

#### 2.1.3 Stage 2: interpretation of analysis

Using standardised RT-qPCR method, the expression norms of our biomarkers were previously established in a fertile cohort. In particular, we documented that an immune profile was reproducible from one cycle to the next over a six-month period if no surgery or pregnancy had occurred in the interim.

Endometrial immune profiles was classified into four types:

1. A balanced endometrial immune activation profile, which is characterised by IL-18/TWEAK and IL-15/Fn-14 mRNA ratios and a CD56+ cell count within the same range as previously defined in the fertile cohort. This profile suggested that the endometrium was ready to go through the following steps of implantation, including apposition, adhesion, and invasion Patients presenting with this endotype were not randomised and excluded from the study.

The three other subgroups represented patients with immune dysregulation who were randomised via the electronic server (Cleanweb- APHP).

2. An under-activated endometrial immune profile was defined by low IL-15/Fn-14 (reflecting immature uNK cells) and/or low IL-18/TWEAK mRNA ratios as well as low CD56+ cell expression.

This profile suggested thatthe endometrium was not fully effective for adhesion and promoting adequate immunotrophism during initial placentation.

3. An over-activated endometrial immune profile was characterised by high IL-18/TWEAK and/or IL-15/Fn-14 mRNA ratios and/or a high CD56+ cell expression.

4. A mixed endometrial immune profile was distinguished by a high IL-18/TWEAK (excess Th-1 cytokines) mRNA ratio and a low IL-15/Fn-14 mRNA ratio (reflecting immature NK cells) and/or low CD56+ expression.

For over-activated and mixed profiles, their profiles suggested that the endometrium were not prepared for the crucial step of trophoblast invasion and may be in a state that can reject the embryo because of a cytotoxic activation of uNK cells in LAKs (lymphocyte-activated killer cells) (30). A test under therapy (glucocorticoids or intralipids) was proposed if the patient was randomised in the personalised arm.

A report, describing the presence or absence of endometrial immune dysregulation was generated and included in the patient’s medical file.

#### 2.1.4 Stage 3: randomisation

Randomisation by blocks was made using the electronic server (Cleanweb- APHP) which allocated patients in a 1:1 ratio to the groups “dysregulated - conventional care” or “dysregulated - precision care” once histological and immune results confirmed the mid-luteal phase and the validity of the endometrial immune profile. Only patients with diagnosed endometrial immune dysregulation were randomised.

If the patient has been randomised for precision care, the report described the suggested treatment plan to apply for the embryo transfer.

##### 2.1.4.1 In case of randomisation: “dysregulated - conventional care”

The patient had a standard fresh or frozen embryo transfer without scratching, or adjunction of corticoids, intralipids, chorionic gonadotropins or double sequential embryo transfer. If the attempt fails, the clinician could decide to personalize the patient’s attempt at the second embryo transfer when the patient ended her participation in the present study.

##### 2.1.4.2 In case of randomisation: “dysregulated - precision care”

Once randomised to the Precision Care group, the treatment they received depended on their individual immune profile- For patients diagnosed with under-active immune profile: the treatment strategy was directed to stimulate mobilisation of immune cells and expression of adhesion molecules.

The precision care was characterised.

- by a endometrial scratching in the mild luteal phase of the cycle preceding the embryo transfer. The objective was to trigger the expression of adhesion molecules and interleukin-15 (31–34).
- by supplementing with chorionic gonadotropins the luteal phase, to trigger local angiogenesis and uNK cells mobilisation (35, 36).
- by advising to have sexual intercourse after the embryo transfer to stimulate the local mobilisation and expression of immune cells (37, 38).

If the patient is over 35 years old with at least one previous ET failure, a double sequential transfer of one embryo on day 3 and one embryo on day 5 was proposed to stimulate the local embryo-endometrium dialogue before implantation (39).

Micronized progesterone for luteal support was usually prescribed at 200 mg three times a day.

- For patients diagnosed with an over-active immune profile or a mixed profile: the strategy aims to down-regulate the local activity of local immune cells.

In this subgroup, immunosuppressive therapy was introduced, aiming at controlling the dysregulated Th-1/Th-2 ratio evaluated by IL-18/TWEAK mRNA expression levels which were elevated in this subgroup. We previously documented using micro-histoculture endometrial models that a high IL-18/TWEAK ratio revealed an underlying cytotoxic activation of uterine NK cells (30). Glucocorticoids was prescribed as a first line of treatment (40, 41)(42) and slow perfusion of intralipids as a second line of treatment in case of resistance to glucocorticoids (43).

The dose of micronized progesterone for luteal support was increased to 400 mg three times a day for its documented immunosuppressive properties (44, 45).

A cycle test under therapy was proposed to evaluate if corticoids or intralipids were able to normalise the endometrial profile. If the endometrial profile was normalised, then the therapy tested was considered as efficient and added for the next embryo transfer. If the endometrial profile was not normalised under corticoids, intralipids was used. If the endometrial profile was not normalised under intralipids, corticoids was used.

If the over-activated profile was associated with, a low uNK cell mobilisation (<10/field) or immature uNK cells (mixed profile), endometrial scratching was added to the cycle preceding embryo transfer and chorionic gonadotropins was used in the luteal phase after the transfer cycle.

Of note, if the patient did not want to have a test of her sensitivity to corticoids, corticoids or intralipids was administered by default.

##### 2.1.4.3 Treatments suggested

Regarding the immune profile, intralipids or/and corticoids and/or chorionic gonadotropins were administered with a variable dosage.

- Glucocorticoid tablets (20 mg daily) was taken by the patient from the third day of the cycle until the pregnancy test and continued for 2 months if pregnant (from 21 days to 3 months) after the endometrial immune analysis. Glucocorticoids was gradually weaned off and stopped in case of negative pregnancy test or after 10 weeks of pregnancy.
- Intralipids (Intralipid 20g/100mL diluted in 400mL of NaCl 0.9%) was administered by slow perfusion during ovarian stimulation (around Day 8 of the cycle) and repeated if pregnant, at 5 weeks and 9 weeks.
- 250µg/0.5 ml of Ovitrelle was administered by subcutaneous route in the luteal phase, 4, 6 and 8 days after the egg collection or the introduction of progesterone (46). This treatment was not prescribed if more than 11 oocytes have been collected or if the oestradiol blood levels on the day of triggering the ovulation was over 3000 pg/ml to avoid ovarian hyperstimulation syndrome.

#### 2.1.5 Stage 4: preparation for the embryo transfer

IVF after a monitored ovarian hyper-stimulation for a fresh embryo transfer as well as endometrial preparation for frozen embryos were conducted as per common protocols.

The delay between the last endometrial immune analysis and the embryo transfer must not exceed 9 months for the mITT analysis. If a spontaneous pregnancy or a gynaecological surgery occured between the biopsy and embryo transfer, patients were excluded from the mITT analysis.

Before 2018, embryo culture until day-5 was not applied as a first-line policy of transfer and day 2-3 embryos were mainly transferred. For the initial transfer, one day-3 embryo was used if the patient was below 30 years old, but in the majority of cases, two day-3 embryos were transferred.

After 2018, the embryo transfer policy has been to favour prolonged culture of embryos until day-5 to promote singletons and prevent multiple pregnancies. Day-3 transfers were only performed if less than 2 embryos were available on day 3 or in case of previous prolonged culture failure.

Endometrial immune profiling did not impact the classical embryo transfer policy except for patients with under-active immune profiles. For these patients, a specific policy of transfer was in place, if they were over 35 years old or previously failed with standard embryo transfer. In such a profile, a sequential double transfer was proposed, the first embryo was transferred on day 2-3 and the second one on day 5-6.

To evaluate the impact of embryo quality on subsequent pregnancy rate, embryo transfers were organised into 2 classes (“top” transfer or “no top” transfer) according to the embryologist’s observations on the day of transfer (47, 48). Each embryo transfer included in the study has been classified anonymously by two distinct embryologists (LR, GC).

On days 2-3, the standard BLEFCO classification (49) was used to evaluate the embryos and on day-5 the Gardner classification (47) was used for the evaluation of blastocyst quality. On day 2-3, “top” grade A high quality embryo was defined as an embryo with typically equal-sized blastomeres or unequal-sized blastomeres according to the number of cells with less than 10% fragmentation. On day-2 the embryo should have 2 to 4 cells, and 6 to 10 on day-3. On day-5, “top” grade A excellent quality Blastocyst was defined as a blastocyst with large, fully expanded blastocoel, inner cell mass, and trophectoderm tightly packed and clearly defined [B5AA-B5AB-B5BA-B4AA-B4AB-B4BA].

Top transfers were defined as the transfer of top quality embryos. If two embryos were transferred, the two embryos have been evaluated as “top”. All the other combinations were classified as “no top” transfer.

Patients for whom a decision to transform the IVF attempt to IUI was taken were included in the mITT analysis as the ITT has been applied and patients were equally represented among the groups. This decision was made because the ovarian response to stimulation was too low (less than 3 follicles) to decide to retrieve oocytes, despite potential fertility on both the male and female side.

#### 2.1.6 Stage 5: outcomes, analysis of the embryo transfer attempts

The live birth rate was defined by the birth of a living baby and was the primary outcome of the mITT analysis. Secondary outcomes were the ongoing pregnancy rate, the clinical pregnancy rate and the miscarriage rate.

The ongoing pregnancy rate was defined by a scan attesting the presence of a gestational sac with an embryonic cardiac activity, that had progressed beyond the first trimester (12 weeks) and was continuing. The clinical pregnancy rate was defined by a BhCG over 100 IU/l in the serum 12 to 10 days after the embryo transfer. Miscarriage referred to the loss of a pregnancy that had occurred after embryo transfer, at any stage of pregnancy, from implantation to the end of the first trimester (12 weeks gestation). Miscarriage did not include biochemical pregnancies that were considered as no pregnancy in our analysis but included early pregnancy losses (gestational sac seen on ultrasound but no heartbeat).

### 2.2 Statistical methodology

Categorical data were presented as numbers (percentages). Continuous variables were presented as means with standard deviations (SDs) and medians with interquartile ranges (interquartile range IQR described as 25th and 75th percentile) for normal and skewed distributions, respectively.

The primary analysis was based on the modified intention-to-treat population (excluding patients without ET) and the primary efficacy endpoint was the live birth rate. The primary efficacy endpoint was compared between the personalised and conventional care using a binary logistic regression.

A logistic regression model was performed including prior known risk factors as covariates (age class, embryo quality, embryo transfer and endometrial immune profile). The 95% two-sided CI for odds ratio (OR) was computed using the bias-corrected and accelerated (BCa) bootstrap interval, OR was presented together with a two-sided 95% BCa confidence interval and associated p-values.

All secondary analyses were based on the mITT population. The secondary binary endpoints were analysed using the same methods as the primary endpoint. For secondary continuous endpoints that were normally distributed with a homogeneity of variance across groups, a t-test was used. For secondary continuous endpoints that were normally or asymptotically normally distributed and heteroscedastic, the Welch t-test was used. For secondary continuous endpoints that are heavy-tailed and skewed, the Mann–Whitney U test was used.

Pre-specified subgroup analyses to evaluate variations in treatment effect were done using logistic regression models, with terms for treatment, subgroup, and interaction of treatment with subgroup. All reported subgroup analyses were pre-specified.

Assuming a 25% birth rate per embryo transfer with conventional care and a 40% relative increase in birth rate with precision care, a sample size of 152 patients per group was needed to achieve 80% power to detect this difference using a chi-square test at a two-sided 5% significance level. Given an anticipated 25% exclusion rate post-randomisation, a total of 380 dysregulated patients (190 per group) had to be randomised. To reach this target, approximately 500 patients were screened since 20% were expected not to be dysregulated. All statistical tests were two-sided and were performed at the 0.05 level. All tests were performed using SAS version 9.4 or later.

## 3 Results

### 3.1 Flow-chart of the study participants

**Figure 2:**
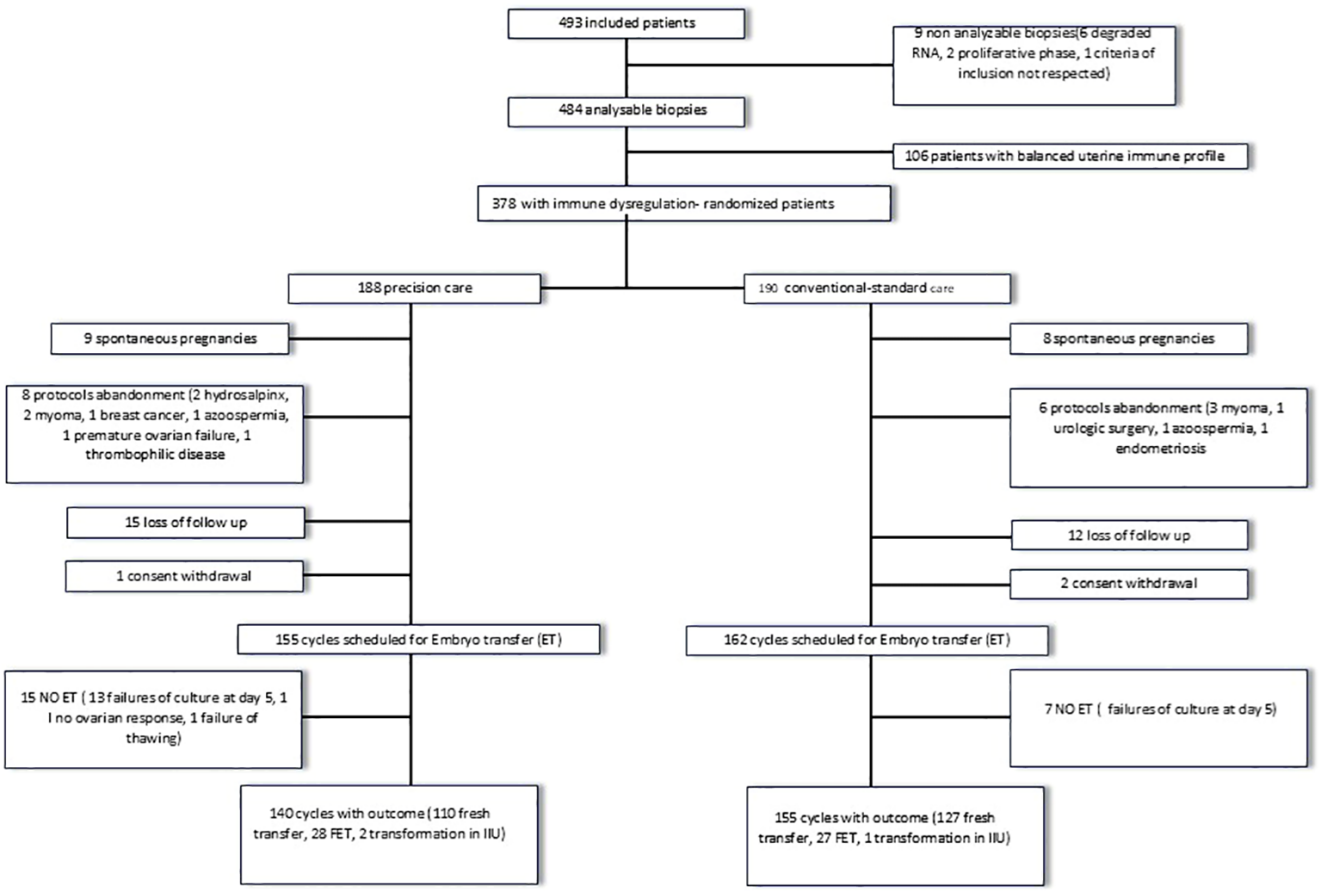
Flow chart of the study.

The **Figure 2** illustrates how patients progressed through the trial. 493 patients were included in this study from October 30th, 2015 to February 8th, 2023. The immune profiling analysis, however, was successfully performed only for 484 patients.

Out of the 484 patients, 78% (378) had a dysregulated endometrial immune profile.

Among deregulated patients, 190 were randomised to receive conventional treatment, and 188 were randomised to receive precision treatment according to their type of immune dysregulation.

14 patients gave up the protocol before the embryo transfer. In 8 cases, surgery was indicated before the embryo transfer and one patient developed endometritis, invalidating the endometrial immune profiling. 3 patients developed azoospermia or ovarian premature failure (exclusion criteria) and 2 couples postponed IVF due to significant health issue. 27 patients were lost to follow-up and did not contact the IVF unit for their embryo transfer after having performed the endometrial immune profiling and 3 patients withdrew their consent.

Finally, 17 patients became pregnant spontaneously. These patients will be treated separately in the analysis because some of these pregnancies seemed to be the direct consequence of identified and treated endometrial immune dysregulation.

Overall, 317 patients have been scheduled for an embryo transfer.

No embryo transfer could be performed in 22 patients (failure of embryo culture until day-5 in 20 cases, no ovarian response in 1 case, failure of embryo thawing in 1 case).

An outcome was available for 295 dysregulated patients: 140 dysregulated with precision care and 155 dysregulated with conventional care.

240 patients were scheduled for a fresh embryo transfer, and among those, 3 were converted to intra-uterine insemination. 58 patients were scheduled for a frozen embryo transfer.

For fresh ET, 80% were stimulated using an antagonist protocol and 20% a long agonist protocol. For frozen transfers, 42% were prepared through natural cycles, 10% with FSH mild stimulation and 8% were prepared with a substituted cycle.

### 3.2 Demographic characteristics, aetiology and past history of study participants

**Table 1A** summarises the clinical and demographic data of patients randomised in conventional versus precision care.

**Table 1A:** Descriptive clinical data of patients included in this study.

	Total N=295	Precision Care N=140	Conventional Care N=155
Age, years			
n (miss.)	295 (0)	140 (0)	155 (0)
Mean ± sd	33.3 ± 3.3	33.5 ± 3.4	33.2 ± 3.2
Median (Q1;Q3)	33.5 (31.1;36.1)	33.7 (31.5;36.1)	33.4 (31.0;36.1)
Min, Max	23.1, 38.7	23.1, 38.7	24.4, 38.5
Age, class — no. (%)			
≥ 35	104 (35.3%)	54 (38.6%)	50 (32.3%)
< 35	191 (64.7%)	86 (61.4%)	105 (67.7%)
All	295 (100.0%)	140 (100.0%)	155 (100.0%)
**AMH ng/ml**	3.16 (2.27;4.70)	3.30 (2.40;4.49)	2.95 (2.21;4.86)
Previous ET failure, two levels — no. (%)			
At least 1 ET failure	197 (66.8%)	95 (67.9%)	102 (65.8%)
No previous ET	98 (33.2%)	45 (32.1%)	53 (34.2%)
All	295 (100.0%)	140 (100.0%)	155 (100.0%)
Previous ET failure, three levels — no. (%)			
Two or more transfer failed	133 (45.1%)	69 (49.3%)	64 (41.3%)
One transfer failed	64 (21.7%)	26 (18.6%)	38 (24.5%)
No previous ET	98 (33.2%)	45 (32.1%)	53 (34.2%)
All	295 (100.0%)	140 (100.0%)	155 (100.0%)
Number of previous embryos transferred			
n (miss.)	295 (0)	140 (0)	155 (0)
Mean ± sd	1.4 ± 1.3	1.5 ± 1.3	1.3 ± 1.2
Median (Q1;Q3)	1 (0;2)	1 (0;2)	1 (0;2)
Min, Max	0, 5	0, 5	0, 5

**Table 1B:** Summary of type of immune imbalance and type of embryo transfer.

	Total N=295	Precision Care N=l40	Conventional Care N=l55
Uterine immune profile - no. (%)			
Over activation	145/295 (49.2%)	711140 (50.7%)	74/155 (47.7%)
Under activation	111/295 (37.6%)	46/140 (32 .9%)	65/155 (41.9%)
Mixt	39/295 (13.2%)	23/140 (16.4%)	161155 (10.3%)
All	295/295 (100.0%)	140/140 (100.0%)	155/ 155 (100.0%)
Number of ET			
n (miss.)	291 (4)	138 (2)	153 (2)
Mean ± sd	1.3 ± 0.5	1.3 ± 0.5	1.2 ± 0.4
Median (Ql ;Q3)	1 (1;2)	1 (1;2)	1 (1;1)
Min, Max	1, 3	1, 3	1, 2
All	290/290 (100.0%)	137/ 137 (100.0%)	153/153 (100.0%)
Stage and number of ET - no. (%)			
1 Day 2/3	(0.68%)	1/138 (0.73%)	1/153 (0.65%)
2 Day 2/3	*521290* (17.9%)	26/138 (18.9%)	261153 (16.9%)
1 Day 5/6	*2061290* (71%)	89/ 138 (64%)	117/153 (76%)
2 Day 5/6	16/290 (5.5%)	7/ 138 (5.1%)	9/153 (5.8%)
DSET	15/290 (5.1%)	15/ 138 (10.9%)	0 (0%)
All	*2901290* (100.0%)	138 (100.0%)	153 (100.0%)
Quality of ET - no. (%)			
No Top	190/291 (65.3%)	91/138 (65.9%)	99/153 (64.7%)
Top	101/291 (34.7%)	47/ 138 (34.1%)	54/153 (35 .3%)
All	291/291 (100.0%)	138/ 138 (100.0%)	153/153 (100%)
Embryo transfer - no. (%)			
Fresh Transfer	222/290 (76.6%)	103/ 137 (75.2%)	119/ 153 (77.8%)
Fet	68/290 (23.4%)	34/ 137 (24.8%)	34/153 (22.2%)
All	290/290 (100.0%)	137/ 137 (100.0%)	153/ 153 (100.0%)

The mean age of the cohort was 33 years old. 64.7% were below 35 years old and 35.3% were over 35 years old.

The main cause of infertility among dysregulated patients was male infertility in 36%, a tubal-related pathology in 20%, an ovulatory problem in 14%, endometriosis in 10%, idiopathic in 10% and recurrent miscarriages in 1%. 9% of the infertility was mixed with male and female factors.

At the time of inclusion, 33% (98/295) never had oocyte retrieval, 50.5% (149/295) failed to be pregnant despite 1 oocyte pick-up and 16% (48/295) failed to be pregnant despite 2 oocyte pick-ups.

Of the 295 patients randomised for whom an outcome was available, 33.2% (98/295) never had any ET before, 21.7% (64/295) previously failed one ET, 27.7% (82/295) previously failed two ET and 17.3% (51/295) failed more than 2 ET (3-5).

According to the new definition of repeated implantation failures (RIF) edited by the ESHRE committee (50) in 2023, 5% (15/295) of the patients randomised with outcome could have been classified as RIF patients in this cohort.

### 3.3 Endometrial immune profiling among study participants

106 patients had a balanced endometrial immune profile, comprising 22% of the cohort, while 378 patients had dysregulated profile, making up 78% of the cohort. No significant differences were observed between the conventional and precision groups with regard to age, previous embryo transfers, fresh or frozen transfers, protocols used, transfer quality, or the distribution of different immune profiles (**Tables 1A, B**). Nor did they differ between dysregulated and non-dysregulated women.

Among dysregulated patients, 30% had under-active profiles, 47% had over-active profiles, and 13.8% had mixed profiles (**Table 1B**).

Therapy testing (glucocorticoids or intralipids) was suggested for patients with overactivation or mixed profiles in the precision group. 83 patients underwent a therapy testing prior ET to verify the immunosuppressive efficacy of the treatment on the diagnosed dysregulation. After testing, 26 patients received glucocorticoids, 44 patients received intralipids (resistance to GC), and 18 received combined intralipids and corticoids. 14 patients with over-activation declined therapy testing, with 10 having the transfer under glucocorticoids and 4 under intralipids. 5 patients with mixed profiles declined testing and received glucocorticoids with additional support.

No side effects or significant adverse events were in the present cohort related to glucocorticoids, slow perfusion of intralipids, or HCG supplementation.

### 3.4 Live birth rates in dysregulated patients randomised to precision versus conventional care

Comparing the LBR between dysregulated patients randomised to conventional versus personalised care constituted the primary endpoint of this study. The modified intention-to-treat (mITT) analysis revealed a significant increase in LBR with precision medical care, rising from 29.7% to 41.4%. The unadjusted odds ratio (OR) was 1.68 [1.04-2.73], p=0.036. Notably, the OR adjusted for age class, embryo quality at transfer, fresh or frozen transfer, and endometrial immune profile type was 1.75 [1.04-2.92], p=0.03).

In terms of the secondary endpoints assessed in the mITT analysis, both clinical pregnancy and ongoing pregnancy rates were consistently elevated with precision care (50.7% and 41.4%, p=0.04) when compared to conventional care (39.4% and 30.4%), as demonstrated through both unadjusted and adjusted analyses. No difference was observed regarding the miscarriage rate between conventional and precision groups. mITT analysis with primary and secondary endpoints are summarised in **Table 2**.

**Table 2:** Primary and Secondary EndPoints, ITT population.

	Total N=295	Precision Care N=140	Conventional Care N=155	Absolute Difference* (95% CI)	Odds Ratio* (95% CI)	P-value^£^	Absolute adjusted Difference** (95% CI)	Odds Ratio** (95% CI)	P-value^££^
**Live Birth rate**	104/295 (35.3%)	58/140 (41.4%)	46/155 (29.7%)	11.8 (1.0 ; 22.6 )^b^	1.68 (1.04 to 2.73)^b^	0.036	11.6 (0.4 ; 22.2 )^b^	1.75 (1.04; 2.92)^b^	0.03
**Ongoing pregnancy rate**	105/295 (35.6%)	58/140 (41.4%)	47/155 (30.3%)	11.1 (0.40 ; 22.0 )^b^	1.63 (1.01; 2.65)^b^	0.048	11.0 (-0.07 ; 21.7 )^b^	1.68 (1.00; 2.81)^b^	0.043
**Clinical pregnancy rate**	132/295 (44.7%)	71/140 (50.7%)	61/155 (39.4%)	11.4 (-0.05 ; 22.5 )^b^	1.59 (1.00; 2.52)^b^	0.052	11.6 (-0.08 ; 22.7 )^b^	1.66 (0.99; 2.70)^b^	0.041
**Early miscarriage rate**	27/133 (20.3%)	13/71 (18.3%)	14/62 (22.6%)	-4.27 (-18.3 ; 9.43 )^b^	0.77 (0.31; 1.87)^b^	0.543	-2.34 (-17.6 ; 11.9 )^b^	0.80 (0.31; 2.04)^b^	0.608

Values are no. (%) or no./total no. (%). CI denotes confidence interval.

*Unadjusted model.

**Adjusted model for Age class, Embryos quality, embryo transfer and Uterine immune profile.

^£^Unadjusted pvalue from Type 3 analysis.

^££^Adjusted pvalue from Type 3 analysis.

^b^Bias-Corrected and Accelerated bootstrap (BCa) confidence interval from 10,000 replications.

**Figure 3:**
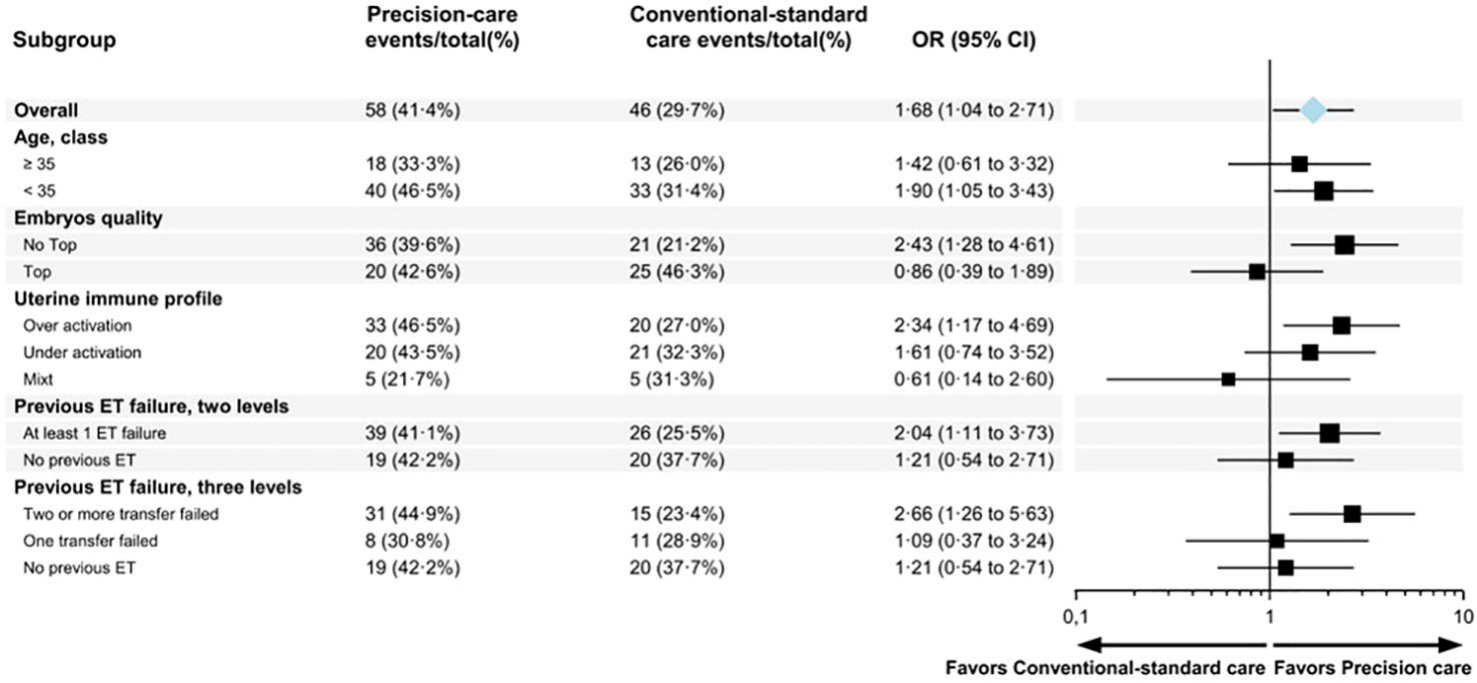
Forest plots describing the odds ratio of live birth comparing precision vs conventional-standard cares.

Subgroup analyses unveiled two particular subgroups which experienced substantial benefits from precision care (**Figure 3**). Firstly, patients with morphologically sub-optimal embryos for transfer (“no top” transfer) exhibited a significant increase in their LBR with precision care (21.2% with conventional care versus 39.6% with precision care, OR: 2.43 [1.28-4.61]). Conversely, for embryos with optimal morphology, immune dysregulation had no discernible impact (46% with conventional care, 43% with precision care).

Furthermore, the subgroup of patients who had previously undergone two or more embryo transfers and experienced failures also significantly benefited from personalisation, resulting in a substantial increase in their LBR (25.5% with conventional care versus 41% with precision care, OR: 2.66 [1.26-5.73]).

In terms of the specific type of immune dysregulation, it appeared that patients with either immune over-activation or immune under-activation benefitted from precision care, while those with a mixed profile did not show the same level of improvement.

Patients with an overactive immune profile who received precision therapy had a significant increase in LBR compared to those who received conventional standard therapy (47% versus 27%, odds ratio [OR]: 2.34 [1.2-4.8]). Notably, the differences were even more pronounced when focussing on patients who underwent the therapy test to assess their sensitivity to the immunosuppressive agent (51% versus 27%, OR: 2.8 [1.34-5.8]). Among patients with both over-active and mixed profiles, those with the therapy test had a significantly higher LBR than those without the therapy test (41% versus 27.7%, p=0.01).

For patients with immune under-activation, the live birth rate (LBR) showed a non-significant increase with precision care compared to conventional care (43% vs. 32%; OR: 1.61 [0.74–3.5], p = 0.23). However, it is essential to highlight as described below that within this subgroup, 8% of patients achieved spontaneous pregnancy after the biopsy, indicating an additional factor contributing to successful outcomes.

### 3.5 Spontaneous pregnancies occurring before the embryo transfer and related to the endometrial immune profile

As the endometrial biopsy performed to collect the endometrium for the endometrial immune profiling is an equivalent of the scratching recommended in an under-activated immune profile to trigger the maturation of uNK by stimulating IL-15 local expression (25), 8% (9 patients) with under-immune activation had a natural pregnancy after the biopsy. 8% (5/64) were randomised in the conventional group and 8% (4/45) were randomised in the precision group among which 87.5% successfully delivered and one had a miscarriage 4 spontaneous pregnancies occurred in the precision group after the test under therapy, 2 resulted in miscarriage and 2 in live birth. The two miscarriages occurred in cases showing an absence of normalisation of the profile under therapy.

2 patients, one over-activated and one with a mixed immune profile included in the conventional group, became spontaneously pregnant and had a miscarriage.

## 4 Discussion

The findings of the present study are novel and of paramount importance in terms of potential implications for routine ART practice. Because of their importance, the caveats and limitations of our study are detailed separately below. In the past, precision interventions targeting the endometrial immune dysregulation were usually dedicated to patients with a history of repeated implantation failure (RIF) and Recurrent Miscarriages (RM) (28). The present study has revealed that an unbalanced endometrial environment may be a significant contributing factor in certain instances of IVF failure among women experiencing infertility, even in the absence of a history of RIF. It is noteworthy that even in this relatively favourable prognosis group undergoing IVF, the proportion and pattern of endometrial immune dysregulation closely resembled those observed in patients with RIF, with 78% showing such dysregulations. This considerable percentage indicates that the endometrial immune profile may not necessarily indicate a pathological condition in the uterus, but may alternatively suggest a less receptive immune environment for implantation. A “less receptive environment” refers to inadequate uterine immune preparation that may impede successful embryo attachment and implantation if the embryo cannot independently correct the local imbalance. To our knowledge, documenting uterine immune equilibrium remains the only method to detect such imbalances, as no tools currently exist to assess immune expression on the embryo’s side. The fact that 30% of patients, despite having an endometrial immune dysregulation, successfully delivered after ET with conventional care provides additional support for this hypothesis. The impact of endometrial immune dysregulation appeared to be negligible when the embryo exhibits optimal morphological quality probably because of its ability to trigger adhesion or control the activation of immune cells if necessary. However, for most patients who have experienced two or more previous implantation failures or have embryos with suboptimal morphology (as noted in 65% of the reported cases), precision treatment may become essential to improve success rates. There is compelling evidence that the endometrium acts as a biosensor, selecting embryos based on their quality for implantation (51). While the embryo itself has the capacity to correct any identified dysregulations, the endometrium may play a pivotal role in rescuing some embryos and enabling their successful implantation. For decades, there has been an ongoing debate about establishing a threshold for the number of previously transferred embryos to define cases of RIF (50, 52). This threshold was the starting point to trigger further research, in particular investigations on the endometrium. This study suggests that the integration of endometrial immune profiling should be taken into consideration at an earlier stage in the patient’s treatment journey. Defining RIF without taking into account the endometrial immune environment and its immune dysregulation as a parameter overlooks a crucial piece of the puzzle.

This study underscores the importance of accurately identifying the specific type of dysregulation through endometrial immune profiling, which in turn determines the most appropriate treatment option. Endometrial immune profiling can provide a basis to identify the most appropriate additional therapies to address local immune dysregulation. The absence of an established immune diagnosis makes the evaluation of specific procedures, such as the random use of steroids or endometrial scratching (53–55) almost impossible. As shown with scratching, a procedure based on immune diagnosis can favour pregnancy, even spontaneous pregnancy (56). In this RCT, 6.8% of infertile patients scheduled for IVF with under-activation became spontaneously pregnant after endometrial biopsy (equivalent to scratching), while only 2.8% of the non-deregulated group became spontaneously pregnant. In contrast, in patients with overactivation, spontaneous pregnancies occurred only after an effective test under treatment, while the other half resulted in miscarriage, suggesting a probable negative effect of the biopsy.

Another effective aspect of our study is the establishment of an endometrial diagnosis coupled with treatment testing to document drug resistance or sensitivity. In most cases, simple, well-known interventions are sufficient to re-establish the local immune balance. However, we also report some cases of corticosteroid resistance, where perfusion of intralipids may have promising outcomes. Clearly, a one-size-fits-all approach is inadequate in the context of infertility, underlining the necessity for precision medicine. It is imperative to use an integrated model considering both the embryo and the endometrium to enhance overall the overall outcomes. The observation that an endometrial immune dysregulation has no impact on pregnancy rates when embryos with optimal morphological features are transferred indicates that a competent embryo is capable of effectively regulating endometrial dysregulation independently. These findings are consistent with previous research indicating that a competent embryo releases pro-adhesive molecules to initiate adhesion (57) and produces immunosuppressive agents to prevent rejection ( (58, 59). Nevertheless, in numerous instances of infertility, the embryos produced may not be fully competent from an immune standpoint. Consequently, addressing diagnosed endometrial dysregulation could exert beneficial effects. As observed by Leese et al., an optimal range of metabolic activity on the embryonic side – referred to as the ‘Goldilocks zone’ – is crucial for maximizing embryonic developmental potential (60, 61). Similarly, it is plausible that a corresponding ‘immune Goldilocks zone’ of regulation may exist on the maternal side as well. Pregnancy relies on a delicate early immune dialogue between the embryo and the endometrium, a process that remains not fully understood (62). The endometrial immune environment, often overlooked in routine clinical practice, is emerging as a crucial factor in improving ART outcomes. Documenting the endometrial immune environment before IVF is straightforward but requires advance planning. It involves collecting a mid-luteal phase endometrial sample via aspiration and analysing it using a patented semi-automated method for precise biomarker quantification. This approach, which complements efforts to enhance embryo quality, could benefit most of patients. Further investigations are needed to evaluate its relevance for routine use, even before IVF. Rebalancing the local immune environment might also facilitate natural conception. Overall, 5% of patients could potentially avoid costly ART through simple, well-known treatment options.

## 5 Limitations

The data presented in this study should be interpreted with caution due to inherent structural limitations that are almost impossible to avoid in human IVF trials. This study was an open-label study with a modified trial design because we had to revise our approach. During the eight years of recruitment, there were several changes in IVF practice, such as a shift from cleaved embryo transfer to blastocyst transfer and the introduction of the ‘freeze-all’ strategy. As a result, the eligibility criteria were expanded to maintain adequate statistical power and to adapt the randomised clinical trial to these changing practices. The single-centre design has its advantages and disadvantages: it minimises variability in practice over time, but also limits the generalisability of the results to other settings. Since this RCT mixed Day-3, Day-5, single or doble embryo transfer, the clear influence of the endometrial environment on the embryo itself also need further investigation. For that reason, we decided to launch a pair matched trial selecting the population who exclusively received a single Day-5 embryo transfer (SET) and benefitted of a uterine immune profiling. This population will be matched to a population. who did not have uterine immune profiling prior to the single Day 5 embryo transfer (NCT06503952). The strength and applicability of our findings depend on replication by independent research teams with an independent validation of these biomarkers, possibly using techniques such as preimplantation genetic testing for aneuploidy (PGT-A) to ensure optimal embryo quality. In addition, replication of these results in patients with RIF and older populations requires careful consideration of potential confounding factors, such as the ploidy of transferred embryos. Conducting randomised controlled trials in the context of IVF failure is essential but challenging, as patients with repeated and unexplained previous failures are often reluctant to participate in randomisation. One possible approach could be to focus on patients who have failed only two embryo transfers. In terms of uterine immune profiling itself, the lack of immune profiling for other key immune cells such as macrophages and dendritic cells and T regulatory cells is a limitation, as these cells are also known to be critical in the implantation process. Moreover, quantifying the individual impact of each of these factors is hard, and assessing their combined effect is even more complex.

## 6 Conclusion

In conclusion, this extended open randomised controlled trial showed that 78% of a standard infertile population undergoing IVF had an immune imbalance in the endometrium at the predicted time of implantation, as identified by endometrial immune profiling. Rebalancing the immune environment with precision therapies led to a significant increase in live birth rates, particularly in patients with previous implantation failures or morphologically sub-optimal embryos. This study provides new evidence for reproductive immunology - a field largely overlooked and underestimated in reproductive medicine - by highlighting the potentially promising role of immune tolerance development in successful pregnancy.

## Data Availability

The datasets presented in this study can be found in online repositories. The names of the repository/repositories and accession number(s) can be found below: NCT02262117- Data supporting the findings of this study will be made available upon reasonable request.
